# Advances in Type 1 Diabetes Mellitus Management in Children

**DOI:** 10.7759/cureus.67377

**Published:** 2024-08-21

**Authors:** Mridu Bahal, Vineeta Pande, Jasleen Dua, Shailaja Mane

**Affiliations:** 1 Pediatrics, Dr. D. Y. Patil Medical College, Hospital and Research Center, Dr. D. Y. Patil Vidyapeeth (Deemed to be University), Pune, IND

**Keywords:** diabetes research, diabetes type 1, endocrinology and diabetes, pediatric diabetes, type i diabetes mellitus

## Abstract

Recent advancements in the management of type 1 diabetes mellitus (T1DM) have significantly improved outcomes and quality of life for patients, particularly children. Technological innovations, such as continuous glucose monitoring (CGM) systems and insulin pump therapy, including hybrid closed-loop systems, have enhanced glycemic control by providing real-time data and automated insulin delivery. Ultrarapid-acting insulins and adjunctive pharmacotherapies, like sodium-glucose transport protein 2 (SGLT2) inhibitors and glucagon-like peptide 1 (GLP-1) receptor agonists, offer improved postprandial glucose management and reduced insulin requirements. Immunotherapy and beta-cell replacement therapies, including stem cell research and encapsulation devices, aim to preserve or restore endogenous insulin production. Digital health platforms and telemedicine have expanded access to education and support, fostering better self-management. Future directions in precision medicine, artificial intelligence, and microbiome research hold promise for personalized and potentially curative treatments. Collectively, these advances are transforming T1DM management, reducing disease burden, and enhancing the prospects for children with T1DM.

## Introduction and background

Type 1 diabetes mellitus (T1DM) is an autoimmune condition characterized by the destruction of pancreatic beta cells, resulting in an absolute insulin deficiency [1,2]. It predominantly affects children and adolescents, posing significant challenges for both patients and their caregivers [3]. The management of T1DM in children requires a multifaceted approach encompassing medical, technological, psychological, and social strategies to maintain glycemic control, prevent complications, and enhance quality of life.

Over the past decades, there have been remarkable advances in the management of T1DM in children. Innovations in insulin therapy, blood glucose monitoring, and technological aids have significantly improved the lives of young patients [4]. Continuous glucose monitoring (CGM) systems, insulin pumps, and artificial pancreas systems have revolutionized diabetes care, offering better glycemic control and greater flexibility in managing the condition. Additionally, new pharmacological therapies and ongoing research into immunotherapy and regenerative medicine hold promise for future breakthroughs [5]. This article provides a comprehensive literature review of the advances in T1DM management in children, highlighting the latest developments and their impact on clinical practice. We will explore traditional management approaches, the role of new technologies and treatments, and the importance of addressing psychological and social aspects. We will also delve into current research and future directions in the quest to improve outcomes for children living with T1DM.

T1DM is an autoimmune disease where the immune system mistakenly attacks and destroys the insulin-producing beta cells in the pancreas [2]. This destruction leads to an absolute deficiency of insulin, a hormone crucial for regulating blood glucose levels. Without insulin, glucose accumulates in the bloodstream, causing hyperglycemia. Chronic hyperglycemia can result in severe complications, including cardiovascular disease, nephropathy, neuropathy, and retinopathy [6]. The pathogenesis of T1DM involves a complex interplay of genetic, environmental, and immunological factors. Genetic predisposition plays a significant role, with certain human leukocyte antigen (HLA) genotypes conferring increased risk [7]. Environmental triggers, such as viral infections and dietary factors, may initiate the autoimmune process in genetically susceptible individuals [8]. The immune response involves both innate and adaptive immunity, with autoantibodies targeting beta-cell antigens like insulin, glutamic acid decarboxylase 65-kilodalton isoform (GAD65), islet antigen 2 (IA-2), and zinc transporter 8 (ZnT8). T cells, particularly autoreactive CD8+ T cells, play a central role in beta-cell destruction [9].

T1DM is one of the most common chronic diseases in children, with incidence rates varying across different regions. It typically presents in childhood or adolescence, with a peak onset between 4 and 14 years of age [10]. The incidence of T1DM is increasing globally, with an annual rise of approximately 3%-5% in many countries [11]. This increase is attributed to multiple factors, including changes in environmental exposures, lifestyle, and diagnostic practices. The prevalence of T1DM also varies widely by geographic region, with the highest rates observed in Scandinavian countries and the lowest in East Asia. In the United States, the prevalence of T1DM among children and adolescents is approximately 1.25 million, with about 200,000 new cases diagnosed annually [12]. The rising incidence of T1DM highlights the need for effective management strategies and continued research into preventive and therapeutic approaches.

The onset of T1DM in children can be rapid and often dramatic, with symptoms developing over a few weeks to months. Common symptoms include: polyuria (frequent urination), polydipsia (excessive thirst), polyphagia (increased hunger), unintentional weight loss, fatigue and weakness, and blurred vision [13]. In some cases, children may present with diabetic ketoacidosis (DKA), a severe and potentially life-threatening condition characterized by hyperglycemia, ketonemia, metabolic acidosis, and dehydration [14]. DKA requires immediate medical attention and hospitalization. The diagnosis of T1DM is confirmed through blood tests that demonstrate hyperglycemia. Diagnostic criteria include fasting plasma glucose ≥ 126 mg/dL (7.0 mmol/L), random plasma glucose ≥ 200 mg/dL (11.1 mmol/L) with classic symptoms of hyperglycemia, glycated hemoglobin (HbA1c) ≥ 6.5%, and oral glucose tolerance test (OGTT) with two-hour plasma glucose ≥ 200 mg/dL (11.1 mmol/L) [4].

Autoantibody testing is also performed to identify the presence of specific autoantibodies associated with T1DM, such as GAD65, IA-2, and ZnT8. Low levels of C-peptide, a marker of endogenous insulin production, further support the diagnosis of T1DM [15]. Early diagnosis and prompt initiation of treatment are crucial to prevent acute complications and long-term sequelae. Effective management strategies aim to maintain glycemic control, reduce the risk of complications, and improve the overall quality of life for children with T1DM.

## Review

Methods

Search Strategy

We conducted a comprehensive literature search in PubMed, Scopus, and Web of Science databases for studies published between January 2000 and December 2023. Keywords included "Type 1 Diabetes Mellitus," "advancements," "continuous glucose monitoring," "artificial pancreas," "immunotherapy," "beta-cell replacement," and "genetic research."

Inclusion criteria: Original research articles, reviews, and clinical trials and studies focusing on new insights and advancements in T1DM.

Exclusion criteria: Studies not related to T1DM and articles without substantial new data or insights.

Data extraction and analysis: Two independent reviewers extracted data on study characteristics, interventions, outcomes, and key findings. Discrepancies were resolved through discussion.

Review

T1DM is an autoimmune condition characterized by the destruction of pancreatic beta cells, resulting in an absolute insulin deficiency [1,2]. It predominantly affects children and adolescents, posing significant challenges for both patients and their caregivers [3]. The management of T1DM in children requires a multifaceted approach encompassing medical, technological, psychological, and social strategies to maintain glycemic control, prevent complications, and enhance quality of life. Over the past decades, there have been remarkable advances in the management of T1DM in children. Innovations in insulin therapy, blood glucose monitoring, and technological aids have significantly improved the lives of young patients [4]. CGM systems, insulin pumps, and artificial pancreas systems have revolutionized diabetes care, offering better glycemic control and greater flexibility in managing the condition. Additionally, new pharmacological therapies and ongoing research into immunotherapy and regenerative medicine hold promise for future breakthroughs [5]. This article provides a comprehensive review of the advances in T1DM management in children, highlighting the latest developments and their impact on clinical practice. We will explore traditional management approaches, the role of new technologies and treatments, and the importance of addressing psychological and social aspects. We will also delve into current research and future directions in the quest to improve outcomes for children living with T1DM. T1DM is an autoimmune disease where the immune system mistakenly attacks and destroys the insulin-producing beta cells in the pancreas [2]. This destruction leads to an absolute deficiency of insulin, a hormone crucial for regulating blood glucose levels. Without insulin, glucose accumulates in the bloodstream, causing hyperglycemia. Chronic hyperglycemia can result in severe complications, including cardiovascular disease, nephropathy, neuropathy, and retinopathy [6]. The pathogenesis of T1DM involves a complex interplay of genetic, environmental, and immunological factors. Genetic predisposition plays a significant role, with certain HLA genotypes conferring increased risk [7]. Environmental triggers, such as viral infections and dietary factors, may initiate the autoimmune process in genetically susceptible individuals [8]. The immune response involves both innate and adaptive immunity, with autoantibodies targeting beta-cell antigens like insulin, GAD65, IA-2, and ZnT8. T cells, particularly autoreactive CD8+ T (cytotoxic T lymphocytes) cells, play a central role in beta-cell destruction [9]. T1DM is one of the most common chronic diseases in children, with incidence rates varying across different regions. It typically presents in childhood or adolescence, with a peak onset between four and 14 years of age [10]. The incidence of T1DM is increasing globally, with an annual rise of approximately 3%-5% in many countries [11]. This increase is attributed to multiple factors, including changes in environmental exposures, lifestyle, and diagnostic practices. The prevalence of T1DM also varies widely by geographic region, with the highest rates observed in Scandinavian countries and the lowest in East Asia. In the United States, the prevalence of T1DM among children and adolescents is approximately 1.25 million, with about 200,000 new cases diagnosed annually [12]. The rising incidence of T1DM highlights the need for effective management strategies and continued research into preventive and therapeutic approaches. The onset of T1DM in children can be rapid and often dramatic, with symptoms developing over a few weeks to months. Common symptoms include polyuria (frequent urination), polydipsia (excessive thirst), polyphagia (increased hunger), unintentional weight loss, fatigue and weakness, and blurred vision [13]. In some cases, children may present with DKA, a severe and potentially life-threatening condition characterized by hyperglycemia, ketonemia, metabolic acidosis, and dehydration [14]. DKA requires immediate medical attention and hospitalization. The diagnosis of T1DM is confirmed through blood tests that demonstrate hyperglycemia. Diagnostic criteria include fasting plasma glucose ≥ 126 mg/dL (7.0 mmol/L), random plasma glucose ≥ 200 mg/dL (11.1 mmol/L) with classic symptoms of hyperglycemia, HbA1c ≥ 6.5%, and OGTT with two-hour plasma glucose ≥ 200 mg/dL (11.1 mmol/L) [4]. Autoantibody testing is also performed to identify the presence of specific autoantibodies associated with T1DM, such as GAD65, IA-2, and ZnT8. Low levels of C-peptide, a marker of endogenous insulin production, further support the diagnosis of T1DM [15]. Early diagnosis and prompt initiation of treatment are crucial to prevent acute complications and long-term sequelae. Effective management strategies aim to maintain glycemic control, reduce the risk of complications, and improve the overall quality of life for children with T1DM.

Results

Traditional Management Approaches

Insulin therapy: Insulin therapy is the cornerstone of T1DM management. It involves the exogenous administration of insulin to compensate for the lack of endogenous insulin production [16]. The goal of insulin therapy is to mimic physiological insulin secretion, maintain blood glucose levels within a target range, and prevent both hyperglycemia and hypoglycemia [17]. Insulins are categorized based on their onset, peak, and duration of action. The main types of insulin used in T1DM management include rapid-acting insulin: insulin lispro, insulin aspart, and insulin glulisine. These insulins have a quick onset (10-30 minutes), peak within 30-90 minutes, and last for 3-5 hours. They are typically administered before meals to control postprandial glucose excursions [18]. Short-acting insulin: Regular insulin falls into this category. It has an onset of 30-60 minutes, peaks at 2-4 hours, and lasts for 5-8 hours. It is also used to control postprandial glucose but has a longer duration than rapid-acting insulins [19]. Intermediate-acting insulin: Neutral protamine Hagedorn (NPH) insulin is an example. It has an onset of 1-2 hours, peaks at 4-12 hours, and lasts for 12-18 hours. It is often used to provide basal insulin coverage [20]. Long-acting insulin includes insulin glargine, insulin detemir, and insulin degludec. These insulins have a prolonged duration of action, lasting up to 24 hours or more, with no pronounced peak. They are used to provide a steady basal insulin level [21]. Insulin can be administered through multiple daily injections (MDI) or continuous subcutaneous insulin infusion (CSII) using an insulin pump. The choice of method depends on the child's lifestyle, preferences, and ability to manage their condition [22]. MDI: This method involves the administration of basal insulin once or twice daily and rapid-acting insulin before meals and snacks. MDI requires frequent blood glucose monitoring and carbohydrate counting to adjust insulin doses [1]. CSII: Insulin pumps deliver insulin continuously through a catheter placed under the skin. Pumps can provide both basal and bolus insulin, allowing for more precise insulin delivery and flexibility in meal timing and activity levels [1]. They also have the advantage of reducing the number of daily injections. Regular blood glucose monitoring is essential to adjust insulin doses and maintain glycemic control. Traditional methods involve fingerstick blood samples measured using a glucometer. The frequency of monitoring depends on the child's age, activity level, and overall health. Self-monitoring of blood glucose (SMBG): SMBG involves pricking the finger to obtain a blood sample, which is then analyzed using a glucometer. Children with T1DM typically need to check their blood glucose levels multiple times a day, including before meals, snacks, and bedtime, as well as during periods of physical activity or illness. HbA1c reflects the average blood glucose levels over the past 2-3 months. Regular HbA1c testing, usually every 3-6 months, is used to assess long-term glycemic control and guide treatment adjustments.

Diet and exercise: A balanced diet and regular physical activity are critical components of T1DM management. Carbohydrate counting helps in adjusting insulin doses according to food intake. Exercise enhances insulin sensitivity and improves overall health, but it requires careful planning to avoid hypoglycemia [23].Diet: Nutritional management focuses on achieving a balanced intake of carbohydrates, proteins, and fats, along with adequate vitamins and minerals. Carbohydrate counting is a key strategy, where the amount of insulin administered is matched to the carbohydrate content of meals and snacks. Regular meals and snacks help maintain stable blood glucose levels [2,23]. Exercise: Physical activity is encouraged as it improves cardiovascular health, insulin sensitivity, and overall well-being. However, exercise can affect blood glucose levels, and adjustments in insulin doses and carbohydrate intake may be necessary to prevent hypoglycemia. Monitoring blood glucose before, during, and after exercise is essential [23]. Despite the availability of effective insulin therapies and monitoring tools, managing T1DM in children presents several challenges. Variability in insulin requirements and insulin needs can vary significantly due to factors such as growth spurts, puberty, illness, and physical activity. Adjusting insulin doses to match these changing requirements is complex [24]. Glycemic variability: Children with T1DM often experience fluctuations in blood glucose levels, making it difficult to achieve consistent glycemic control. Hypoglycemia and hyperglycemia pose significant risks [25]. Adhering to the rigorous demands of insulin administration, blood glucose monitoring, and dietary adjustments can be challenging for children and their families. The chronic nature of T1DM and the constant need for management can lead to emotional stress, anxiety, and burnout for both patients and caregivers. Providing psychological support and counseling is essential to address these issues [26]. 

Technological Advances in T1DM Management

CGM: CGM systems have revolutionized the management of T1DM by providing real-time glucose readings and trends. These systems offer significant advantages over traditional fingerstick methods, allowing for better glycemic control and reducing the risk of hypo- and hyperglycemia [27]. CGM systems consist of three main components: (1) Sensor: A small sensor inserted under the skin measures glucose levels in the interstitial fluid. The sensor typically needs to be replaced every 7-14 days, depending on the model. (2) Transmitter: The transmitter attaches to the sensor and sends glucose data to a receiver or a compatible device, such as a smartphone or insulin pump. (3) Receiver/display device: The receiver or display device shows real-time glucose readings, trends, and alerts for high and low blood glucose levels. CGM systems provide continuous glucose readings, typically updated every five minutes. They also offer trend information, which helps in understanding how glucose levels change over time and in response to various factors like food intake, physical activity, and insulin administration. CGM systems offer significant benefits in the management of diabetes. They provide detailed, real-time information on glucose levels and trends, which allows for more precise insulin dosing and better overall glycemic control. By continuously monitoring glucose levels, CGM systems help prevent hypoglycemia by alerting users to impending low blood sugar levels, enabling timely interventions and reducing the risk of severe episodes. Studies have also demonstrated that CGM use is associated with lower HbA1c levels over time, indicating improved long-term glycemic control. Moreover, CGM reduces the need for frequent fingerstick tests, which is particularly advantageous for children and enhances the quality of life for both patients and caregivers by providing peace of mind. However, CGM systems do have limitations that need consideration. Cost is a significant barrier for many families, as these systems can be expensive, and insurance coverage may vary. Additionally, some CGM models require periodic calibration with fingerstick tests, which can be inconvenient and disrupt continuous monitoring. While CGM sensors are generally accurate, there can be discrepancies between interstitial glucose measurements and actual blood glucose levels, especially during rapid glucose changes. Moreover, the insertion of sensors can cause skin irritation or discomfort for some users, impacting their comfort and compliance with continuous use [28].

Insulin pumps: Insulin pumps have significantly improved the management of T1DM by offering more precise insulin delivery and greater flexibility compared to MDIs [29]. There are two main types of insulin pumps. (1) Tethered pumps are connected to the body by tubing. The pump itself is typically worn on a belt or in a pocket. Examples include Medtronic MiniMed and Animas pumps. (2) Patch pumps adhere directly to the skin and do not use tubing. They are controlled by a handheld device or a smartphone app. Examples include the OmniPod system [30]. Insulin pumps offer precise insulin delivery by providing continuous basal insulin and adjustable bolus doses at mealtimes, closely mimicking physiological insulin secretion compared to injections. This precision allows for greater flexibility in meal timing and activity levels, enabling users to easily adjust insulin delivery according to changes in diet, exercise, and daily routines. Such flexibility helps reduce the risk of hypoglycemia, particularly at night, by fine-tuning insulin administration. Additionally, many patients report an improved quality of life with insulin pumps, as they alleviate the burden of MDIs and provide more stable glucose control. However, there are challenges associated with insulin pumps. The cost of the pumps and their supplies can be prohibitive, with insurance coverage varying significantly. Regular maintenance is required, including changing the infusion site every 2-3 days and replacing batteries, which can be cumbersome. Technical issues, such as pump malfunctions or infusion set blockages, may also arise, necessitating prompt resolution to maintain effective glucose management. Moreover, the effective use of insulin pumps demands thorough training and continuous support from healthcare providers to ensure optimal outcomes.

Artificial pancreas systems: Artificial pancreas systems, also known as closed-loop systems, represent a significant advancement in T1DM management. These systems combine CGM and insulin pump technologies with advanced algorithms to automate insulin delivery, reducing the burden of diabetes management [31]. The artificial pancreas system is designed to closely mimic the body's natural glucose regulation and consists of three primary components: a CGM, an insulin pump, and a control algorithm. The CGM continuously monitors glucose levels in real-time, providing essential data for insulin management. This data is then utilized by the insulin pump, which delivers insulin doses based on the real-time glucose readings from the CGM. At the heart of the system is a sophisticated control algorithm. This algorithm processes the CGM data and adjusts insulin delivery dynamically to maintain glucose levels within a target range. By automatically regulating insulin delivery based on CGM, the artificial pancreas system aims to reduce the need for manual adjustments and enhance glycemic control. Overall, artificial pancreas systems offer a more precise and responsive approach to managing blood glucose levels compared to traditional methods. This automated adjustment of insulin delivery can significantly improve glycemic control, thereby potentially improving the quality of life for individuals with diabetes. Numerous clinical trials have demonstrated the safety and efficacy of artificial pancreas systems. Key findings from these trials include [32,33] the following: Artificial pancreas systems have shown remarkable benefits in improving glycemic control and enhancing patients' quality of life. These systems effectively increase time in range (TIR), reduce HbA1c levels, and decrease the incidence of hypoglycemia, ensuring more stable glucose levels and minimizing diabetes-related complications. Patients using these systems report a significantly better quality of life, experiencing reduced anxiety about glucose control and enjoying greater flexibility in their daily activities. Moreover, the automatic insulin delivery adjustments provided by these systems eliminate the constant need for manual monitoring and intervention. Several artificial pancreas systems have received regulatory approval, confirming their safety and efficacy and making them widely available for clinical use. This advancement represents a significant leap forward in diabetes management, offering patients a more seamless and effective means of maintaining optimal glucose levels. Examples include the Medtronic MiniMed 670G and the Tandem Control-IQ system [34]. The development of artificial pancreas systems is continually advancing, with research focused on improving accuracy, reducing device size, and integrating additional hormones like glucagon. Future systems may offer fully automated, multi-hormonal control, further enhancing glycemic outcomes and easing diabetes management. Meanwhile, the rise of mobile apps and digital tools has provided invaluable resources for managing T1DM, offering real-time glucose monitoring, insulin dosage calculations, and improved communication between patients and healthcare providers. These innovations collectively promise a brighter future for diabetes management. These tools offer features such as blood glucose tracking, insulin dose calculation, food and exercise logging, and data sharing with healthcare providers [35]. Several effective apps have emerged for T1DM management, such as MySugr, which tracks blood glucose levels and calculates insulin doses, and integrates with CGM devices. Glooko syncs data from various diabetes devices to offer glucose trend insights and allows healthcare providers to monitor progress. The Dexcom G6 App, tailored for the Dexcom G6 CGM system, delivers real-time glucose readings, alerts, and trend information [36].

Pharmacological Advances

Pharmacological advances in T1DM management have introduced new insulin formulations and adjunctive therapies, enhancing glycemic control and reducing complications [37]. Ultrarapid-acting insulins, such as faster-acting aspart (Fiasp) and ultrarapid Lispro (Lyumjev), offer quicker onset and shorter duration, closely mimicking natural insulin responses to meals. Long-acting insulins like Iisulin degludec (Tresiba) and insulin glargine U300 (Toujeo) provide stable, prolonged coverage with lower hypoglycemia risk [38]. Adjunctive therapies, including SGLT-2 inhibitors and GLP-1 receptor agonists, offer additional glycemic control benefits, weight loss, and potential cardiovascular and renal protection, providing comprehensive tools for managing T1DM.

Ongoing research continues to explore new pharmacological treatments for T1DM, aiming to improve outcomes and reduce the burden of disease management. Dual agonists, which simultaneously target GLP-1 and glucose-dependent insulinotropic polypeptide (GIP) receptors, are being investigated for their potential to provide superior glycemic control and weight loss compared to existing therapies [39]. These agents may offer a novel approach to T1DM management by enhancing multiple pathways involved in glucose regulation. Insulin-IgG fusion proteins are a novel class of long-acting insulins designed to extend the half-life of insulin and provide more stable glucose control. These fusion proteins combine insulin with the Fc region of IgG, resulting in prolonged circulation time and reduced dosing frequency [40]. Encapsulation technologies aim to protect transplanted beta cells from immune attack while allowing the cells to function and secrete insulin. Encapsulated beta-cell therapies could offer a potential cure for T1DM by restoring endogenous insulin production without the need for immunosuppression [41].

Psychological and Social Aspects of T1DM Management

T1DM significantly affects the lives of children and their families, impacting daily routines, emotional well-being, and overall quality of life. The chronic nature of the disease, coupled with the constant need for monitoring and management, can be overwhelming and stressful for both patients and their caregivers.

Children with T1DM may experience a range of emotional and psychological challenges, including [42] the fear of hypoglycemia and hyperglycemia, along with concerns about long-term complications, can lead to heightened anxiety levels. The constant burden of managing a chronic illness may contribute to feelings of sadness, hopelessness, and depression. Diabetes burnout is another critical issue, where the relentless demands of self-management and adherence to treatment regimens result in fatigue and frustration. Additionally, social isolation can occur as children worry about being different from their peers and struggle to manage diabetes in social settings, leading to a sense of exclusion and loneliness. Psychological support and counseling are vital for children with T1DM and their families. These services help manage the emotional challenges of the condition, offering a safe space to express feelings and reduce stress. They teach coping strategies to handle the emotional burden, improve adherence to treatment, and address behavioral issues. Family counseling enhances communication, fosters a supportive environment, and guides parents in supporting their child's emotional well-being, all contributing to better overall diabetes management.

Advances in Immunotherapy and Beta-Cell Regeneration

Ongoing research into immunotherapy and beta-cell regeneration holds promise for potentially altering the course of T1DM and providing a functional cure [43].

Immunotherapy: Immunotherapy aims to halt the autoimmune destruction of pancreatic beta cells, thereby preserving or restoring endogenous insulin production. Anti-CD3 monoclonal antibodies like teplizumab target CD3 molecules on T cells, modulating the immune response and preserving beta-cell function. Clinical trials have shown that anti-CD3 antibodies can delay the onset of T1DM in high-risk individuals and preserve residual beta-cell function in newly diagnosed patients [44]. Anti- interleukin-2 (IL-2) receptor antibodies like basiliximab and other anti-IL-2 receptor antibodies inhibit T cell proliferation, reducing the autoimmune attack on beta cells. These agents are being tested in combination with other immunotherapies to enhance their efficacy [45]. Antigen-specific therapies aim to induce immune tolerance to specific beta-cell antigens, such as insulin or GAD65, thereby preventing autoimmune destruction. Strategies include peptide vaccines, DNA vaccines, and nanoparticle-based delivery systems [46].

Beta-cell regeneration: Beta-cell regeneration focuses on restoring the body's ability to produce insulin by regenerating or replacing lost beta cells. Key approaches include stem cell therapy which researchers are exploring the use of pluripotent stem cells to generate functional beta cells. Methods involve differentiating stem cells into insulin-producing cells and transplanting them into patients. Ongoing trials are assessing the safety and efficacy of encapsulated stem cell-derived beta cells to protect them from immune attack [47]. Scientists are investigating beta-cell proliferation that stimulate the proliferation of existing beta cells. Growth factors, such as betatrophin, have shown potential in promoting beta-cell replication in animal models. Transdifferentiation involves reprogramming other cell types, such as alpha cells or ductal cells, into insulin-producing beta cells. Gene editing technologies like CRISPR-associated protein 9 (CRISPR-Cas9) are being utilized to induce transdifferentiation and restore beta-cell function.

Stem cell therapy: Stem cell therapy [47] offers a promising avenue for T1DM treatment by potentially restoring endogenous insulin production through beta-cell regeneration. Pluripotent stem cells, such as embryonic stem cells (ESCs) and induced pluripotent stem cells (iPSCs), can differentiate into insulin-producing beta cells. Significant advancements include the development of differentiation protocols that efficiently convert pluripotent stem cells into functional beta cells and encapsulation technologies that protect transplanted beta cells from immune attacks while allowing nutrient and insulin exchange. Clinical trials are currently assessing the safety and efficacy of these encapsulated stem cell-derived beta cells in T1DM patients. Mesenchymal stem cells (MSCs) are being studied for their potential in treating T1DM due to their immunomodulatory properties and ability to promote tissue repair. They secrete anti-inflammatory cytokines and growth factors, which can reduce autoimmune attacks and support beta-cell survival. Researchers are also exploring the combination of MSCs with other regenerative therapies, like beta-cell transplantation or immunotherapy, to enhance treatment outcomes.

Genetic Research and Precision Medicine

Advancements in genetic research and precision medicine are paving the way for personalized T1DM management tailored to individual genetic profiles and disease characteristics. Genetic research, including genome-wide association studies (GWAS) and single-cell genomics [48], is uncovering mechanisms of T1DM and identifying potential therapeutic targets. GWAS have pinpointed genetic variants linked to T1DM susceptibility, aiding the development of targeted therapies, while single-cell technologies reveal the heterogeneity of immune cell populations involved in T1DM, informing more effective immunotherapy designs [49]. Precision medicine aims to tailor treatments based on an individual's genetic makeup, disease phenotype, and response to therapy. Biomarker discovery is guiding personalized treatment plans by predicting disease progression, treatment response, and risk of complications. Additionally, targeted therapies are being developed to address specific genetic and molecular pathways in T1DM, enhancing efficacy and minimizing side effects [50].

Pediatric Maturity Onset Diabetes of the Young (MODY)

Pediatric MODY is a rare, inherited form of diabetes that typically manifests in adolescence or early adulthood. Unlike type 1 diabetes, which is autoimmune in nature, or type 2 diabetes, which is often associated with lifestyle factors, MODY is caused by mutations in specific genes involved in insulin production [51]. There are several types of MODY, each linked to different genetic mutations. The most common genes involved include hepatocyte nuclear factor 1-alpha (HNF1A), hepatocyte nuclear factor 4-alpha (HNF4A), and glucokinase (GCK) [51,52]. Genomic interventions for MODY focus on identifying and correcting the genetic mutations responsible for the disease. Advances in genomic technologies have enabled the precise editing of genes, offering the potential for curative therapies. The primary approaches includes gene editing techniques such as CRISPR-Cas9 have revolutionized the ability to directly modify DNA. For MODY, this could involve correcting the specific mutations in genes like HNF1A or GCK, restoring normal insulin production and function [53,54]. Gene therapy involves introducing a correct copy of the mutated gene into the patient's cells. Viral vectors are commonly used to deliver the functional gene, potentially curing the condition by compensating for the defective gene [53]. RNA interference (RNAi) and antisense oligonucleotides (ASOs) can be employed to target and degrade mutant mRNA transcripts, reducing the expression of the defective protein and mitigating the disease's impact [54].

Beyond direct genomic interventions, several reformative strategies can enhance the management and treatment of MODY [55]. Genetic testing can identify the specific type of MODY, allowing for tailored treatment plans. For example, patients with HNF1A mutations often respond well to sulfonylureas, a class of oral hypoglycemic agents [52]. CGM systems can provide real-time insights into blood sugar levels, helping to maintain optimal glucose control and prevent complications. Educating patients and their families about MODY, its genetic basis, and management strategies can improve adherence to treatment and quality of life. Ongoing research into the molecular mechanisms of MODY can uncover new therapeutic targets and lead to the development of novel interventions. Pediatric MODY is a genetically driven form of diabetes that presents unique challenges and opportunities. Genomic interventions and personalized treatment strategies hold promise for effective management and potential cures, transforming the outlook for affected individuals [56].

Innovative Technologies in Development

Emerging technologies hold promise for further advancing T1DM management, offering more precise and convenient options for patients. Smart insulin pens are designed to improve insulin administration by providing features such as dose tracking, reminders, and data sharing [57]. Smart pens record the time and amount of each insulin dose, helping patients and healthcare providers monitor adherence and adjust treatment plans. These devices can send reminders for insulin administration and alert users to potential missed doses, enhancing adherence. Smart pens can sync with mobile apps and digital platforms, allowing for seamless integration of insulin data with glucose monitoring systems.

Implantable glucose sensors offer a long-term solution for CGM, reducing the need for frequent sensor changes. Eversense® E3 CGM: The Eversense system features an implantable sensor that lasts up to 180 days, providing continuous glucose data with fewer sensor replacements. The system includes a wearable transmitter that communicates with a smartphone app [58]. Researchers are developing next-generation implantable sensors with enhanced accuracy, longer lifespan, and improved biocompatibility.

Advanced closed-loop systems, or hybrid artificial pancreas systems, aim to automate insulin delivery more effectively by integrating multiple hormones and incorporating advanced algorithms. These systems combine insulin and glucagon delivery to better mimic the body's natural glucose regulation. The addition of glucagon can help prevent hypoglycemia and improve overall glucose control. Advanced algorithms use machine learning and predictive analytics to optimize insulin delivery in real-time, adapting to individual patterns and minimizing glycemic variability.

Potential future breakthroughs

Ongoing research and development efforts are focused on achieving significant breakthroughs in T1DM management, with the potential to transform the landscape of diabetes care. Fully automated artificial pancreas systems aim to achieve complete automation of glucose regulation, eliminating the need for manual adjustments.AI-driven algorithms can enhance the performance of artificial pancreas systems by learning from individual glucose patterns and predicting future glucose trends. Future systems may integrate with wearable devices, such as fitness trackers and smartwatches, to incorporate additional physiological data and improve glucose control. Beta-cell replacement therapies, including stem cell-derived beta cells and islet transplantation, offer the potential for restoring endogenous insulin production [59]. Encapsulated stem cell-derived beta cells protect transplanted beta cells from immune attack, offering a potential cure without the need for immunosuppression. Advances in islet transplantation techniques, including the use of immunomodulatory agents and novel delivery methods, are improving the success rates and long-term outcomes of this approach. Innovative drug delivery methods aim to enhance the efficacy and convenience of T1DM treatments. Researchers are developing oral insulin formulations that can withstand the digestive process and deliver insulin effectively via the gastrointestinal tract. Inhalable insulin devices, such as Afrezza, offer a noninvasive alternative to injections, providing rapid-acting insulin for mealtime glucose control [60,61].

## Conclusions

The management of T1DM in children has seen remarkable advancements driven by technological innovations, new pharmacological therapies, and a deeper understanding of the disease. CGM systems, insulin pumps, and artificial pancreas systems have revolutionized diabetes care, providing better glycemic control and improved quality of life. New insulin formulations and adjunctive therapies offer additional tools to optimize treatment. Equally important are the psychological and social aspects of managing T1DM, which require comprehensive support and resources to help children and their families navigate the challenges of the condition. Ongoing research into immunotherapy, beta-cell regeneration, and stem cell therapy holds promise for future breakthroughs that could transform the landscape of T1DM management. As we look to the future, continued investment in research, innovation, and support systems is essential to further improve outcomes and enhance the lives of children living with T1DM. The collective efforts of healthcare providers, researchers, families, and communities will pave the way for a brighter future for young patients with T1DM.
